# Supplementary material for: Are carbon emissions trading and green financial instruments synergistic? -Comprehensive quantitative research based on content analysis
Source: PLoS One. 2024 Mar 7;19(3):e0298601. doi: 10.1371/journal.pone.0298601 (PMC10919601; doi:10.1371/journal.pone.0298601)
Supplement: S1 Data — (ZIP) [file pone.0298601.s001.zip › Data/地级市Z_F工作报告.zip!/║╙─╧/╫ñ┬φ╡Ω╩╨/└┤╫╘╣1⁄2╓┌║┼í╛┬φ┐╦╩2╛▌═°í┐_2004.docx]

各位代表：

　　现在，我代表市人民政府，向大会作政府工作报告，请予审议，并请各位政协委员提出意见。

　　一、2003年政府工作情况

　　2003年，市人民政府在省委、省政府和市委的领导下，在市人大、市政协的监督和支持下，团结带领全市人民，开拓进取，扎实工作，较好地完成了市一届人大四次会议确定的各项任务。

　　(一)国民经济继续保持增长态势。全年完成国内生产总值345.7亿元，比上年增长1.8%，其中第一产业下降17.3%，二、三产业分别增长12.2%和9.9%；全年完成工业增加值128.2亿元，增长11.9%，其中限额以上工业增长14.6%，综合效益指数为118.4%；社会消费品零售总额127.7亿元，增长10.5%；地方财政一般预算收入完成11.95亿元，为年度调整预算的101.2%，财政支出完成33亿元，增长8%；城镇居民人均可支配收入5676元，增长7.03%；农民人均纯收入1726元，下降17%。

　　(二)实施项目带动战略成效显著。全年完成全社会固定资产投资86.6亿元，增长13.3%。年初确定的60个重点项目完成投资38.96亿元，占年度计划的95%。其中竣工投产项目16个，完成投资22.5亿元；续建项目7个，完成投资3.08亿元；计划新开工及预开工项目29个，已开工26个，开工率90%，完成投资12.4亿元。西气东输豫南支线、古城电厂1×6万千瓦机组、天方药业欧典螺旋霉素技改、卷烟厂技改、驻新路驻马店至汝南段、开龚路上蔡至汝南段等项目相继建成投运；蓝天集团年产30万吨甲醇、吴桂桥煤矿、古城电厂2×30万千瓦机组、骏马集团精细化工园、豫龙水泥日产5000吨熟料生产线以及白云纸业填平补齐等项目已开工建设，在建项目数量和投资规模均创历史最高水平。

　　(三)改革开放迈出新的步伐。国企改革力度加大，全市140户拟改制企业中，有124户完成了改制任务，其他16户企业正在改制之中。积极推进行政审批制度改革，全年取消、合并、下放行政审批事项194项，市行政服务中心建成启用。农村税费改革继续深化，政府机构改革稳步推进，经济社会发展的体制环境进一步改善。民营经济快速发展，全市新发展个体工商户1.3万户，新登记注册私营企业357户；全年实现增加值、利润均比上年增长40%以上，民营经济上缴税金占全市税收总额的43%。深入开展东西合作，成功举办了第六次东西合作经贸洽谈会，全年共履约东西合作项目525个，引进市外资金32.9亿元，项目履约率和资金到位率分别为77.5%和45.6%。积极开展对外贸易，全市出口创汇完成3226万美元，增长16.5%，利用外商直接投资1518万美元，增长22.8%。

　　(四)结构调整取得新的进展。农业优质产品比重提高，优质小麦、优质玉米面积分别增长47.3%和24.5%，食用菌、花生、蔬菜、花木、中药材等种植面积扩大，农业生产的区域化、规模化特点更加鲜明；订单农业和标准农业稳步发展，已建立无公害农产品基地29个。林业生产成效显著，全年完成成片造林40.78万亩，退耕还林23.5万亩，新建、完善农田林网242万亩，完成通道绿化2112公里。优质畜禽养殖规模不断扩大，生产结构进一步优化，肉、蛋、奶产量分别较上年增长7.09%、7%和25.6%，畜牧业产值占农业总产值的比重达到56.02%。工业技改力度加大，全市14项重点工业技改项目完成投资13亿元，增长44%，企业适应市场的能力继续增强。旅游业快速发展，实现旅游总收入1.75亿元，增长26.7%；嵖岈山风景区被评为国家级地质公园、国家4A级景区。金融保险、房地产、现代物流快速发展，餐饮、商贸等传统服务业发展水平进一步提高。

　　(五)城镇化进程进一步加快。市区完成城市建设投资6.33亿元，增长26.6%。练江路立交桥、107国道景观路改造等一批重点工程相继竣工，新区广场、温州步行街和东二环南段、健康路西段、仓库路东段、南海路北段等工程进展顺利，城市供水、供气、电力、通讯等基础设施得到完善。加强城市管理，深入开展城市环境综合整治活动，市容市貌得到改观。加快以县城和29个重点小城镇为骨干的城镇体系建设，全市城镇化水平达到15.02%，中心城区面积近40平方公里。

　　(六)就业再就业和社会保障工作得到加强。认真贯彻落实再就业工作的方针政策，加大资金投入和政策扶持力度，积极搞好再就业服务，千方百计开发再就业岗位。全年新增就业岗位37162个，下岗失业人员累计实现再就业22911人，其中“4050”人员7854名，城镇登记失业率为3 %。认真做好“两个确保”和城市低保工作，共发放国有企业下岗职工基本生活保障金1393.24万元，企业离退休人员养老金22707万元，城市低保金5062万元。深入开展帮扶困难职工活动，帮助5313名下岗职工实现再就业。

　　(七)经受住了非典和自然灾害的严峻考验。面对突如其来的非典疫情，我们沉着应对，把非典防治作为重中之重，建立了五个机制，部署五道防线，落实五项保障，加强疫病监测、疫情控制和医疗救治，取得了无死亡病例、无继发性病例、无医务人员感染的重大胜利。面对多年不遇的严重自然灾害，各级政府全力做好抗灾自救工作。先后下拨救灾款3640万元，帮助灾民重建住房18991间，落实了卫生防疫、开仓借粮等措施，积极开展生产自救，确保了大灾之年灾民有饭吃、有衣穿、有房住、有病能及时医治，无适龄儿童因灾辍学。

　　(八)科技教育和各项社会事业全面发展。科技工作稳步推进，全年共组织实施科技攻关、星火计划、火炬计划等项目308项，科技对经济增长的贡献率进一步提高。教育事业全面发展，“两基”成果得到巩固提高，农村义务教育体制改革顺利实施，教师工资发放、公用经费、危房改造投入保障机制逐步建立。高中教育发展迅速，全市普通高中在校生达到10.9万人，比上年增加1.5万人。职业教育、成人教育、高等教育稳步发展，教育资源整合力度加大，专升本已经国家高校设置评议专家组评议。以疾病预防控制和农村为重点，加强公共卫生体系建设，全年争取卫生专项资金10327万元，基本建设实现了历史性突破；艾滋病防治救助工作取得明显成效，全年各级累计投入资金近5000万元，较好地解决了艾滋病人医治和群众的生产生活困难。高度重视人口和计划生育工作，人口自然增长率5.2‰。环境保护执法力度加大，环境质量得到改善。文化艺术事业进一步繁荣，群众性体育活动健康发展。深入开展军民共建活动，荣获全国“双拥模范城”称号。统计、人事、审计、民族宗教、广播电视、外事侨务、气象、人防、史志等各方面都取得了新的成绩。

　　(九)精神文明和民主法制建设进一步加强。深入开展“双创”活动，城乡面貌和广大干部群众精神风貌发生了新的变化。坚持依法治市，开展普法教育，增强了全市人民的法制观念。自觉接受人大法律监督和政协民主监督，认真办理人大代表议案、建议和政协委员提案。重视群众来信来访工作，妥善处理群众反映的热点、难点问题。健全安全生产管理体制，大力整顿和规范市场经济秩序，深入开展反腐纠风工作，加大社会治安综合治理力度，维护了社会大局稳定。

　　各位代表，2003年是极不寻常的一年，在遭遇非典疫情和严重自然灾害的情况下，我们能够取得这样的成绩，确实来之不易。这些成绩的取得，是省委、省政府和市委正确领导的结果，是全市人民团结奋斗、辛勤劳动的结果。在此，我代表市人民政府向工作在全市各条战线的广大工人、农民、知识分子、干部、驻驻部队指战员、武警官兵、政法干警和社会各界人士致以崇高的敬意！向所有关心支持政府工作的各位人大代表、政协委员和民主党派、工商联、无党派人士表示衷心的感谢！

　　在肯定成绩的同时，我们也清醒地看到，我市发展中还存在不少困难和问题。主要表现在：经济总量小，人均国内生产总值等指标远低于全国、全省平均水平，实现全面建设小康社会目标，任务十分艰巨；农业基础薄弱，抗御自然灾害能力差，农民增收难度大；工业科技含量低，骨干企业少，结构不尽合理，制约因素较多；就业和社会保障任务繁重，城乡不少低收入居民生活还比较困难；财政收支矛盾突出，特别是乡镇财政十分困难；一些地方社会治安问题比较突出，刑事犯罪案件屡有发生；政府廉政建设、经济发展环境和干部作风等仍是群众反映比较强烈的问题。所有这些困难和问题，我们一定高度重视，在今后工作中认真加以解决，决不辜负人民群众的期望和重托。

　　二、2004年政府工作的总体要求和预期目标

　　2004年是深入贯彻党的十六大和十六届三中全会精神、全面建设小康社会的关键一年，也是我市抢抓机遇、加快发展的重要一年。根据中央、省委和市委有关经济工作的安排部署，今年政府工作的总体要求是：坚持以邓小平理论和“三个代表”重要思想为指导，牢固树立全面、协调、可持续的发展观，以全面建设小康社会统揽全局，坚持“二、一、三、四”发展思路，强力实施项目带动战略，大力促进结构调整，继续推进体制创新，进一步优化发展环境，切实转变经济增长方式，着力解决经济社会发展中的突出矛盾，着力解决关系人民群众切身利益的突出问题，努力提高经济增长的质量、效益和人民生活水平，实现国民经济持续快速健康发展和社会全面进步。

　　今年我市经济和社会发展的主要预期目标是：国内生产总值增长11.5%，其中第一产业增长13%，第二产业增长11%，第三产业增长10.5%；全社会固定资产投资增长19.4%；社会消费品零售总额增长9%；外贸出口增长12%；地方财政收入与经济同步增长；城镇居民可支配收入和农民人均纯收入分别增长8%和9%；城市登记失业率控制在4%以内；人口自然增长率控制在5.6‰以内。

　　实现上述目标，尽管存在一些挑战、困难和不确定因素，但也面临多方面的发展机遇和有利条件。从宏观环境看，世界经济和贸易呈现增长趋势，国内消费结构和产业结构升级步伐加快，必将引发新的市场需求扩张；国家更加注重搞好宏观调控和统筹兼顾，并继续实施积极的财政政策和稳健的货币政策，有利于我们欠发达地区争取更多的项目和资金；今年中央一号文件在政策上加大了对“三农”问题的支持力度，有利于我们农业大市发挥优势和挖掘潜力；全省中原崛起战略的实施和结构调整、“三化”进程的加快，也将对我市经济发展产生积极的推动作用。从我市情况看，经过不断探索和总结，确立了一条符合实际的“二一三四”发展思路；经过不断挖掘和开发，内在优势逐渐显现；经过不断培育和扩充，经济总量显著增加，经济发展后劲明显增强；经过不断治理和规范，发展环境逐步优化，干部群众干事创业的热情日益高涨。只要我们抢抓机遇，倍加珍惜来之不易的大好形势；只要我们坚定信心，进一步增强加快发展的紧迫感和使命感;只要我们开拓创新、知难而进、务实苦干，我们的奋斗目标就一定能够实现！ 

　　三、大力实施项目带动战略，努力加快“三化”进程

　　(一)坚持项目带动，努力培育经济发展后劲

　　全力以赴抓好重点项目建设。今年计划安排投资在3000万元以上的重点项目70个，总投资256亿元，年度计划投资66.6亿元，项目数量和投资规模均比上年有大幅度增长。今年安排竣工投产项目22个，总投资40.5亿元，年计划投资24.1亿元，包括豫龙水泥日产5000吨熟料生产线、骏马化工技改、白云纸业年产10万吨文化用纸填平补齐、蓝天集团年产10万吨甲醇、华骏车辆铸造扩建、西气东输豫南支线等8个工业项目；驻信高速驻马店南和确山两条连接线，以及商桐路、确平路、高兰路、君二路等7个交通项目；练江路改造、市中心医院门诊楼等7个其他项目。今年安排续建项目21个，总投资70.8亿元，年计划投资17亿元，包括蓝天集团年产30万吨甲醇、古城电厂2×30万千瓦净化燃烧机组、吴桂桥煤矿、骏马化工集团精细化工园、汝南双星鞋业等6个工业项目；小洪河治理、老王坡滞洪区安全建设等3个农业基本建设项目；九县一区天然气管网建设、热电厂供热管网扩建、会展中心等5个城建项目；驻马店高中分校、市二中分校、驻马店师专扩建等4个社会事业项目，以及岈山、薄山湖、天中山等3个旅游基础设施项目。今年安排计划新开工及预备新开工项目25个，总投资134.8亿元，年计划投资24.9亿元，包括蓝天集团2×35万千瓦燃气电厂、天方药业扩建、西平年产10万吨玉米淀粉糖、麦科特集团镁合金加工等6个工业项目；阿深高速(驻马店段)、上武高速(驻马店段)、新阳高速(新蔡至泌阳)等9个交通项目；新区行政中心、市垃圾处理场等4个城建项目，以及市体育馆、疾病控制中心等建设项目。对于这些重点项目，要千方百计加快建设进度，确保22个计划竣工项目年内建成投运，21个续建项目完成年度形象进度，争取计划新开工及预备新开工项目如期开工建设。

　　做好项目储备和申报工作。认真研究国家有关政策，抓紧论证一批关联度强的大项目、好项目，充实项目库；做好市白云纸业二期扩建工程、豫龙水泥第二条日产5000吨熟料生产线等项目的前期工作，积极创造开工条件。

　　加强项目协调和管理。严格执行国家的产业政策，注重提高项目档次和质量。继续加强项目工作组织领导，健全项目工作机制。采取市场化、多元化、国际化的运作方式多渠道筹措资金，为项目建设提供资金保证。全面落实项目法人责任制、招投标制、建设监理制、工程质量终身责任制，加强监督管理，确保工程进度和质量。

　　(二)走新型工业化道路，加快工业化进程

　　按照新型工业化要求和盘活存量、扩大增量、增加储量的基本思路，牢固树立工业兴市意识，加快结构调整，搞好科技创新，实现工业速度和效益同步增长。拓宽融资渠道，积极争取国债资金、工业结构调整专项资金、高新技术产业化贴息资金、银行贷款，大力吸纳民间投资，切实增加工业投入。努力培育和壮大优势产业，加快传统产业技术改造步伐，支持一批对结构调整有重大带动作用的项目建设，提高产业技术装备水平。突出抓好生物工程、化工等产品的扩能和提升，壮大规模，提高产品质量；利用先进技术改造纺织、食品加工、机械制造等行业，开发产品品种，增强市场竞争力；积极发展高新技术产业，注重发展劳动密集型产业，加强信息资源开发和应用，推进信息化进程。

　　继续实施“名牌战略”。整合工业资源，加快产业聚集，积极引导生产要素向支柱产业和优势企业集中。加速天方药业、骏马化工、蓝天集团、银马纺织、十三香、金雀电气、华骏车辆等骨干企业向拥有知名品牌、主业突出、核心竞争力强的大企业、大集团发展。加大天方罗欣片剂、骏马牌尿素、华骏牌车辆等产品的推介力度，提高品牌知名度。加强对工业经济运行的监测和指导，搞好资源调度，缓解供需矛盾，督促企业落实安全生产措施，确保工业在良好的生产经营环境中运行和发展。

　　(三)开展“双创”活动，加快城镇化进程

　　继续深入开展“双创”活动，突出中心城区建设，加快县城建设，支持重点镇建设，进一步提高城镇化水平。

　　科学做好城镇规划。严格执行中心城区总体规划，认真编制行政新区、工业新区、旧城改造规划以及环卫设施、园林、文化体育设施、城市道路网等专业性、控制性规划。全面完成9个县城的总规修编，加快行政区划近期、中期、远期规划编制工作。加强规划控制与管理，坚持规划一张图、审批一支笔，规范审批程序，加大执法力度，坚决制止违规违法建设行为，坚决维护规划的严肃性和权威性。

　　切实加强城市建设与管理。围绕建设园林城市、生态城市、卫生城市的总体目标，着重抓好水、气、电、暖、通讯、污水及垃圾处理等基础设施和学校、医院等公共服务体系建设，完善城市功能，加强城区生态保护，不断改善人居环境。突出抓好中心城区建设，实施练江路、雪松路改造工程和骏马河治污工程，加快会展中心建设进度，启动行政区北迁和垃圾处理场工程，完成风光路、骏马路景观改造。搞好道路排水和小游园、小绿地建设，提升绿化档次，提高城市绿化覆盖率。大力开展城市环境综合整治，促进城区交通秩序、市场秩序、环境卫生明显好转。强化市民文明意识，增强市民参与城市管理的积极性。推进市政公用事业、建筑企业、勘察设计体制改革步伐，搞活以土地为主的城市资产经营，逐步建立“投资多元化、建设市场化、管理经营化”的新机制，进一步拓宽经营城市路子。

　　大力发展城镇经济。充分发挥比较优势，突出当地特色，培育支柱产业，增强城镇经济实力。加快旅游业发展，统筹规划，完善功能，提升品位，打响“三山两湖”(岈山、铜山、天中山、薄山湖、宿鸭湖)旅游品牌，提高景区知名度。逐步完善城镇住房、就业、医疗、教育和社会保障等制度改革，积极引导农民和农村二、三产业向城镇集中，形成产业发展、人口聚集、市场扩大的良性互动机制，促进城镇可持续发展。

　　(四)围绕增加农民收入，加快农业现代化进程

　　解决农业、农村和农民问题，是我们全部工作的重中之重。今年要按照统筹城乡发展的要求，紧紧围绕增加农民收入这个核心，全面落实中央一号文件精神和党在农村的各项政策，采取更加有效的措施，确保农业农村经济在灾后恢复中实现新的发展。

　　坚持市场导向、因地制宜、示范带动、农民自愿、强化服务的原则，继续调整优化农业结构。稳定粮食种植面积，扩大优质小麦、优质玉米面积，提高粮食生产能力。积极推进优势农产品区域化布局，扩大优势农产品生产规模。进一步做大上蔡花木、正阳花生、确山瓜果和中药材、平舆白芝麻、汝南蔬菜等一批特色农业。加快林果业开发步伐，努力扩大经济林果面积，重点抓好特色杂果种植和开龚路、驻上路、驻新路、107国道两侧花木带建设；加快造林绿化步伐，争取三分之一的县今年达到高级绿化标准。大力发展畜牧养殖业，加快规模养殖基地建设，积极引导和支持产销联建的20家养殖定点企业发展，推进畜牧业集约化生产、规模化经营。大力培育农业产业化龙头企业，特别是农畜产品精深加工企业，积极发展农产品批发市场和农村经济合作组织，加快农业产业化步伐。加强农产品质量标准和安全监测体系建设，推进无公害农业、标准农业、创汇农业和品牌农业的快速发展。

　　加强农业基础设施建设。抓住灾后重建、水毁工程修复和淮河治理的机遇，搞好防洪除涝和兴利工程建设，抓好板桥、宿鸭湖、薄山、宋家场四大灌区的修复和节水改造，加快平原打井步伐，确保全年新增有效灌溉面积30万亩，新增旱涝保收田20万亩。继续开展山、水、林、田、路综合治理，进一步改善农业生态环境。

　　拓宽农民增收渠道。积极组织和引导农民发展农村二、三产业和个体私营经济。加强对农民工的培训，取消对农民工的各种歧视性政策和不合理收费，完善信息网络，搞好劳务输出的组织和管理，引导支持农民外出务工，增加务工收入。采取切实措施，加强监督和协调，认真解决拖欠农民工工资问题。全面落实中央逐步降低农业税税率和对种粮农民实行直接补贴的政策，确保农民群众真正得到实惠。严格落实耕地保护制度，确保土地依法征收征用，及时给予农民合理补偿，切实保护农民合法权益。做好农民负担监管工作，巩固农村税费改革成果，规范“一事一议”，防止农民负担反弹。

　　大力发展县域经济。县域经济是全市经济发展的基础，全面建设小康社会，关键在县域经济，重点和难点也在县域经济，必须把发展县域经济放在更加突出的位置。立足于发挥比较优势、形成区域特色、壮大产业规模，做大做强工业经济，放手发展民营经济，积极培育特色经济，大力提升劳务经济，增强县域经济实力。加大对县域经济发展的政策支持、金融支持的力度，制定切实可行的发展措施，加强科学指导，强化协调服务，优化发展环境，促进县域经济快速发展。

　　四、推进体制改革，扩大对外开放，增强经济发展的动力和活力

　　推进国有企业改革。继续采取产权转让、收购兼并、合资合作、破产重组等形式，吸引外资和民间资本参与国有企业的改组、改造，大力发展混合所有制经济。加强对国有资产的监督管理，在规范操作的前提下，实现国有资本的有序退出。做好改制企业的规范完善工作，加快未改制企业的改革进度，力争年内完成改革任务。加大流通企业改革力度，搞好分类排队，先易后难，积极推进。搞好国有粮食购销企业改革，转换国有粮食企业经营机制，全面放开粮食购销市场。进一步完善企业法人治理结构，分离企业办社会职能。妥善解决改革中职工安置、资产处置、债权债务处理等问题，确保改革健康进行。

　　大力发展民营经济。认真落实促进民营经济发展的各项政策措施，放宽市场准入，降低门槛，鼓励民营经济投资支柱产业、基础产业等领域，参与能源、交通、城市公共设施建设。进一步消除政策歧视，改进对民营企业的服务，加大依法保护力度，确保民营企业在税收、信贷支持、土地使用、对外贸易、经营环境等方面与其他企业享受同等待遇。引导、鼓励和支持民营企业改善管理，规范行为，加快建立现代企业制度步伐，重点扶持顶志、十三香、华强、荣光、大成、鼎力和男儿风等民营企业，使之不断发展壮大。 加快社会信用体系建设。逐步建立信用市场监督管理体系和失信惩戒制度。继续整顿和规范市场秩序，重点抓好直接关系人民群众身体健康和生命安全的食品、药品等方面的专项整治，严厉打击制售假冒伪劣产品和商业欺诈行为。加强银企沟通，搞好项目对接，增加金融机构在我市的贷款投放。规范发展信贷业务，认真落实有关小额贷款担保政策，增加对消费需求的信贷支持。严厉打击逃废金融债务行为，为金融机构创造良好的经营和发展环境。

　　加快推进财税体制改革。实施市直国库集中支付改革，深化政府采购、部门预算、会计集中核算改革，提高财政资金使用效益。强化税收征管，坚持依法治税、依率计征，做到应收尽收。切实加强罚没收入、行政事业性收费、土地使用权和市政设施经营权转让收入等非税收入管理，认真落实“收支两条线”和“票款分离”的规定。严格支出管理，合理调整财政支出结构，控制、压缩一般性支出，确保机关事业单位人员工资、社会保障等重点支出。积极探索、逐步建立财政支出绩效评价体系，切实加强对政府投资的监督管理，规范政府投资行为，健全科学决策机制，提高政府投资效益。

　　继续深入开展东西合作。进一步落实鼓励外来投资的政策规定，突出抓好签约项目履约工作，提高项目履约率和资金到位率。认真落实招商引资责任制，采取多种有效形式，开展招商引资活动，确保招商引资实效。认真总结东西合作的经验，积极探索推动东西合作的新路子，做好2004年全国东西合作会的筹备和组织工作，确保会议圆满成功。

　　进一步扩大对外经济合作与交流。鼓励各类所有制企业开展出口业务，扩大工业制成品和优质农产品出口规模，努力巩固传统出口市场，积极开拓新兴市场。广泛开展对外商务活动，吸引港澳台地区及外国投资。积极探索利用外资的途径，扩大引资领域和利用外资规模。加强跟踪服务和协调，抓好外资项目的实施。支持有条件的企业到境外投资发展，开展不同形式的对外劳务合作。

　　五、统筹兼顾，促进经济社会协调发展

　　坚持以人为本，树立科学的发展观，统筹经济和社会协调发展，是我们必须长期坚持的指导思想，也是解决当前经济社会发展中诸多矛盾必须遵循的基本原则。要实现全面建设小康社会的目标，必须从人民群众切身利益出发，切实做好就业和社会保障工作，坚持不懈实施科教兴市、人才强市和可持续发展战略，大力发展各项社会事业。

　　做好就业和社会保障工作，努力改善人民生活。千方百计增加就业是政府的重要职责。继续实行积极的就业政策，特别要把小额贷款、税费减免等优惠政策落到实处。拓宽就业渠道，注重发展劳动密集型产业、中小企业和社区服务业，推行灵活多样的就业方式，鼓励自主创业和自谋职业。做好就业援助和就业服务，巩固和扩大“帮扶”成果，优先安排“4050”人员到政府开发的公益性岗位就业。力争新增城镇就业33000人以上，下岗失业人员再就业9300人以上。进一步完善社会保障体系。继续做好“两个确保”工作，搞好“三条保障线”的衔接。完善城镇职工基本养老保险、基本医疗保险和失业保险制度，扩大各项社会保险覆盖面。进一步做好城市“低保”工作，规范“低保”标准和范围，做到应保尽保。对城乡特困群众给予更多的关爱，切实帮助特困家庭解决就医看病、子女上学、住房等实际困难。完善农村“五保户”生活保障制度，确保供养资金。高度重视救灾工作，落实灾区税收减免政策，做好受灾群众生产生活救济工作，确保“四有一无”目标的实现。加大扶贫开发工作力度，确保完成年度脱贫任务。

　　加强科技工作，发展教育事业。编制好科技规划，加大科技投入，大力发展高新技术产业，加强企业技术中心建设，抓好先进技术的引进、消化和应用，提高高新技术产业在工业增加值中的比重。大力实施科技兴农，巩固和发展农业科技示范园区，推广农业新技术，促进科技成果的转化。支持高新区加快建设步伐，增强其对资金、人才、技术等要素的吸引聚集效应。积极发展高等教育，继续做好“专升本”工作。整合中等教育资源，抓紧申办驻马店高等职业技术学院，筹建医学专科学校。统筹城乡教育，加强农村教育，重点发展高中阶段教育，积极发展职业教育和成人教育，全面推进素质教育。重视人才工作，开发人才资源，努力营造吸引人才、留住人才、人尽其才的良好氛围。

　　推进卫生、文化、体育等社会事业发展。完善公共卫生体系，加强疾病控制体系、突发公共卫生事件医疗救治体系、重大疫情信息网络体系建设，努力提高对各种疾病及卫生事件的防范和应对能力。突出抓好农村卫生工作，切实加强乡镇卫生院建设，推进乡镇卫生院改革。继续做好农村新型合作医疗制度试点工作。重视艾滋病防治救助工作，逐步建立长效的防治救助机制。大力发展文化事业，抓好文化场馆和广播电视网络等基础设施建设，加强文物保护，推进文化产业发展，繁荣文学艺术、新闻出版、社会科学、广播电视事业。积极开展各类群众性文化体育活动，办好市一届运动会，提高竞技体育水平。加强国防教育，关心、支持驻驻部队和民兵预备役建设，深入开展双拥共建活动，进一步密切军政军民关系。

　　坚持可持续发展战略，促进人口、环境和社会协调发展。加强人口与计划生育工作，稳定低生育水平，确保人口和计划生育目标的实现。加强统计工作，组织好全市第一次经济普查，完善统计制度和方法，提高统计数据的质量和时效。加强国土资源管理，合理开发和综合利用土地、矿产、森林等自然资源，提高资源利用效率。以水污染防治为重点，加大环境保护与污染治理力度，进一步改善生态环境。

　　六、推进精神文明和民主法制建设，保持社会大局稳定

　　加强精神文明建设。继续实施《公民道德建设实施纲要》，深入开展“道德规范进万家、诚实守信万人行”活动，加强社会公德、职业道德和家庭美德教育，提高全民思想道德素质。坚持以创建文明行业、文明单位、文明社区、文明村镇、文明家庭为重点，进一步把精神文明创建活动引向深入。认真组织科技、文化、卫生“三下乡”活动，引导农民普及科技知识，搞好殡葬改革，破除陈规陋习，反对封建迷信，逐步形成文明健康的生活方式。

　　推进民主与法制建设。各级政府要自觉接受人大及其常委会的监督，认真执行人大及其常委会的各项决定和决议，支持人大代表依法行使职权；认真办理人大代表议案、建议和政协委员提案。加强与人民政协的协商，自觉接受人民政协的民主监督；发挥工会、共青团、妇联等人民团体的桥梁、纽带作用。进一步搞好政务、厂务、乡镇和村务公开，完善村民自治和企业民主管理制度。逐步建立和完善重大问题集体决策制度、专家咨询制度、社会公示和听证制度，提高决策的民主性、透明度和公众参与度。加强法制宣传，推进依法治市，不断提高全社会的法制意识。

　　保持社会稳定。保障人民群众安居乐业，是政府应尽的职责。要进一步落实社会治安综合治理各项措施，健全治安防范网络，预防和减少犯罪。始终保持严打态势，坚持依法从重从快严厉打击黑恶势力犯罪、严重暴力犯罪、多发性侵财犯罪，增强人民群众的安全感。坚决依法打击“法轮功”等邪教组织和非法宗教活动。以对国家和人民高度负责的精神，切实做好安全生产工作，健全责任制，落实防范措施，确保人民生命财产安全。全面贯彻党的民族宗教政策，继续做好民族宗教工作。高度重视人民群众来信来访工作，认真调解社会矛盾纠纷，切实解决好群众反映强烈的问题，妥善处置各种群体性事件，化解各种不安定因素，保持社会大局稳定。

　　七、加强政府自身建设，努力提高工作效率和水平

　　实现全面建设小康社会的奋斗目标，全面完成今年的各项工作任务，各级政府和领导干部必须适应新形势、新任务，不断提高行政能力和管理水平。

　　进一步转变政府职能。按照建设“行为规范、运转协调、公正透明、廉洁高效”行政管理体制的要求，切实把政府的职能转移到经济调节、市场监管、社会管理和公共服务上来。继续推行和完善政务公开制、服务承诺制、首问责任制和失职追究制等行之有效的制度，强化对职能部门的监督和约束。推进政企分开，进一步把不该由政府管理的事项交给企业、社会组织和中介机构，充分发挥市场在资源配置中的基础性作用。政府该管的事一定要管好，并适应新形势改进管理方式和方法，提高行政效能和工作效率。

　　坚持依法行政。各级政府都要按照法定权限和程序行使权力、履行职责。所有工作人员都要学会并善于依法处理经济社会事务。以实施《行政许可法》为契机，进一步清理政府规范性文件，切实做好行政许可主体清理工作，继续精简行政审批事项，减化审批程序。认真落实执法责任制和执法过错追究制，加强执法监督，促进严格执法、公正执法和文明执法，不断提高各级政府依法行政水平。

　　牢固树立执政为民思想。政府的一切权力都是人民赋予的，必须对人民负责，为人民谋利益。各级政府一定要把为最大多数人谋利益作为一切工作的出发点和落脚点。制定政策，要充分了解民情，看是否符合群众的意愿；落实政策，要充分依靠群众，求得大多数人的支持。要始终坚持维护群众的利益高于一切、关心群众的疾苦重于一切、解决群众的困难先于一切的原则，真心实意为群众办实事、做好事、解难事，力求把涉及人民利益的每一项工作做好。

　　大兴求真务实之风。弘扬求真务实精神是政府建设的重要内容。我们想问题、办事情、作决策，都要符合本地实际，一切从实际出发，按客观规律办事，讲求工作实效。要树立正确的政绩观，力戒形式主义，不提脱离实际的指标，不喊哗众取宠的口号，不搞劳民伤财的政绩。要健全工作责任制，强化目标管理考核，加大政府督查力度，从严执政，奖罚分明，确保工作落实。各级政府工作人员都要做到廉洁自律，严格遵守反腐倡廉的各项规定，自觉接受党和群众的监督，经受住权力和金钱的考验。要以对党和人民高度负责的态度，保持良好的精神状态，奋发有为，迎难而上，努力在全市营造聚精会神搞建设、一心一意谋发展的良好氛围。

　　各位代表，我们正处在全面建设小康社会的重要阶段，正处在中原崛起和驻马店全面振兴的关键时期，摆在我们面前的任务光荣而又艰巨。让我们在省委、省政府和市委的领导下，在市人大、市政协的监督支持下，以责无旁贷的使命感，以只争朝夕的紧迫感，紧紧依靠全市人民，振奋精神，坚定信心，励精图治，务实苦干，为圆满完成今年的各项任务、实现全面建设小康社会的宏伟目标而努力奋斗！
